# Neural Tube Defects and *ZIC4* Hypomethylation in Relation to Polycyclic Aromatic Hydrocarbon Exposure

**DOI:** 10.3389/fcell.2020.582661

**Published:** 2020-11-16

**Authors:** Yun Huang, Shanshan Lin, Chengrong Wang, Xin Pi, Lei Jin, Zhiwen Li, Linlin Wang, Aiguo Ren

**Affiliations:** ^1^National Health Commission Key Laboratory of Reproductive Health, Department of Epidemiology and Biostatistics, Institute of Reproductive and Child Health, School of Public Health, Peking University Health Science Center, Beijing, China; ^2^Ministry of Education-Shanghai Key Laboratory of Children’s Environmental Health, Xinhua Hospital, Shanghai Jiao Tong University School of Medicine, Shanghai, China; ^3^Division of Birth Cohort Study, Guangzhou Women and Children’s Medical Center, Guangzhou Medical University, Guangzhou, China; ^4^Department of Obstetrics and Gynecology, Guangzhou Women and Children’s Medical Center, Guangzhou Medical University, Guangzhou, China; ^5^Beijing Obstetrics and Gynecology Hospital, Capital Medical University, Beijing, China; ^6^Department of Social Medicine and Health Education, School of Public Health, Peking University, Beijing, China

**Keywords:** neural tube defects, DNA methylation, polycyclic aromatic hydrocarbons, *Zic4*, oxidative stress

## Abstract

**Background:**

Epigenetic dysregulation is one of the postulated underlying mechanisms of neural tube defects (NTDs). Polycyclic aromatic hydrocarbons (PAHs), a group of environmental pollutants that are reported as a risk factor of NTDs, may cause decreased genome-wide DNA methylation. With DNA extracted from neural tissues, this study identified gene(s) whose hypomethylation was related to elevated risk for NTDs and examined whether its hypomethylation is related to PAH exposure.

**Results:**

Using data profiled by Infinium HumanMethylation450 BeadChip array from 10 NTD cases and eight controls, *ZIC4*, *CASP8*, *RAB32*, *RARA*, and *TRAF6* were identified to be the top five genes in NTD-related hypomethylated gene families. Among all identified genes, *ZIC4* had the largest number of differently methylated CpG sites (*n* = 13) in the promoter region and 5′ UTR. Significantly decreased methylation in the *ZIC4* promoter region and 5′ UTR was verified in an independent cohort of 80 cases and 32 controls (*p* < 0.001) utilizing the Sequenom EpiTYPER platform. Hypomethylation of *ZIC4* was associated with a higher risk of NTDs [adjusted OR = 1.08; 95% confidence interval (CI): 1.03, 1.13] in a logistic regression model. Mean methylation levels in the promoter region and 5′ UTR of *ZIC4* tended to be inversely associated with levels of high-molecular-weight PAHs in fetal liver among NTD fetuses (β [95% CI]: −0.045 [−0.091, 0.001], *p* = 0.054). Six and three CpG sites in the *ZIC4* promoter region and 5′ UTR were inversely correlated with antioxidant indicators and protein oxidation markers (*ρ*: −0.45 to −0.75, *p* < 0.05) in fetal neural tissues, respectively. In a whole-embryo cultured mouse model, hypomethylation of the *Zic4* promoter region and 5′ UTR and upregulation of *Zic4* were observed, coupled with increased NTD rates after BaP exposure. The antioxidant *N*-acetyl-L-cysteine normalized the changes observed in the BaP exposure group.

**Conclusion:**

Hypomethylation of the *ZIC4* promoter region and 5′ UTR may increase the risk for NTDs; oxidative stress is likely to play a role in the methylation change of *Zic4* in response to PAH exposure in NTD formation.

## Introduction

Neural tube defects (NTDs), a group of fatal and disabling congenital defects, are caused by the failure of the morphogenetic process of neural tube closure during embryogenesis. NTDs have a multifactorial etiology and are thought to arise from complex gene–environment interactions that remain poorly understood (Copp and Greene, 2010). Epigenetic regulation has been suggested to string fetal development, and DNA methylation is one of the best-characterized epigenetic modifications, which coordinates normal gametogenesis and embryogenesis. Emerging evidence of an association between DNA methylation dysregulation and NTD formation indicates that alterations in fetal methylation may underlie NTD etiology.

The developmental origins of health and disease hypothesis asserts that exposure to environmental pollutants during embryonic development, a critical period for epigenetic reprogramming, leads to epigenetic dysregulation, and results in increased disease risk (Heindel and Vandenberg, 2015). Several previous epidemiological studies support a positive association between maternal polycyclic aromatic hydrocarbon (PAHs) exposure and elevated risk for the development of NTDs in offspring, when PAHs or PAH–DNA adducts were used as exposure markers in maternal serum, placental tissue, cord blood, and cord tissue (Ren et al., 2011; Yuan et al., 2013; Wang et al., 2015; Yi et al., 2015). PAHs are common persistent organic pollutants in the environment formed during incomplete combustion of organic materials. The substances therefore easily reach the fetus by crossing the placenta barrier (Perera et al., 2005; Al-Saleh et al., 2013; Mohammadi et al., 2017). Moreover, decreased genome-wide DNA methylation associated with PAH exposure has been reported in studies of genomic DNA derived from umbilical cord blood cells (Herbstman et al., 2012; Tang et al., 2012). Thus, epigenetic change is postulated to be a possible mechanism through which the interaction between PAH exposure and NTD-related genes is mediated.

Transplacental PAHs can cause oxidative stress (OS), leading to macromolecular oxidative damage. Evidence from human, animal, and cell studies suggests that PAH-induced OS plays a role in DNA methylation dysregulation, especially in DNA hypomethylation (Fan et al., 2012; Lan et al., 2016; Udomsinprasert et al., 2016; Zhu et al., 2017; Huang et al., 2019b). Although several studies have demonstrated that decreased methylation levels in certain genes may relate to NTD formation using a candidate-gene approach (Rochtus et al., 2016; Tian et al., 2018; Zhang et al., 2019; Huang et al., 2019b), no study has ever identified hypomethylated NTD-related genes based on genome-wide DNA methylation analysis. In addition, the mechanisms through which these DNA hypomethylation changes arise are yet to be fully explored.

In this study, we identified hypomethylated genes in NTD cases, utilizing genome-wide DNA methylation data profiled by the Infinium HumanMethylation450 array. The methylation level of the identified gene was validated with the neural tissue of fetuses from an independent cohort of cases and controls. We then evaluated the association between methylation levels in the identified gene and fetal PAH exposure. To gain insight into the possible mechanisms of the observed methylation change, correlations between the methylation levels of CpG sites in the identified gene and concentrations of OS markers in fetuses were further analyzed. Finally, a mouse model was utilized to test the findings from the case–control studies in human subjects, with the methylation and expression of the identified gene being assayed in mouse embryos treated with benzo[a]pyrene (BaP) and embryos co-treated with BaP and antioxidant *N*-acetyl-L-cysteine (NAC).

## Materials and Methods

### Human Subjects

Neural tube defect cases and controls were recruited from Shanxi Province in northern China between 2011 and 2014 (Ren et al., 2011). Epidemiological studies have shown that the prevalence of NTD in this Province is very high, 31.5/10,000 in 2014 (Liu et al., 2016). Cases were terminated fetuses following prenatal diagnosis of an NTD or stillborn infants with an NTD; controls were aborted fetuses due to unintended pregnancies. Experienced pathologists performed the autopsy and collected brain, spinal cord, and liver tissues from cases and controls. All tissue samples were immediately stored at -80°C after collection. Data on sociodemographic characteristics and lifestyle of case and control mothers, including periconceptional folic acid supplementation, were collected with a questionnaire at birth or at pregnancy termination. Written informed consent was obtained from all women. This study was approved by the Institutional Review Board of Peking University (Beijing, China).

### Animals and *in vitro* Whole-Embryo Culture

In this experiment, 2-month-old primigravida CD-1 (ICR) mice were used. The mice were provided food and tap water *ad libitum* and housed in a temperature- and humidity-controlled facility with an ambient temperature of 21 ± 2°C, relative humidity of 50 ± 10%, and a 12-h light/12-h dark cycle. Embryonic day 0 (E0) was designated at first detection of a vaginal plug after the mating period. As described previously (Huang et al., 2019b), mouse embryos were dissected out of the uteri at E8.5. The maternal membranes, decidua, and Reichert’s membrane were removed under sterile conditions in Hank’s medium. Then, the embryos were distributed alternately into culture bottles with rat serum in a gas atmosphere of O_2_, CO_2_, and N_2_. The bottles were then sealed, and embryos were cultured for 48 h at 37°C with 1‰ dimethyl sulfoxide (DMSO), 5 μM BaP or 5 μM BaP, and 5 μM NAC at a rotation speed of 25 rpm. Neural tissue was separated from the E10.5 mouse and stored at −80°C for further analysis. The Institutional Animal Care and Use Committee at Peking University approved all protocols describing the animal research reported here.

### Methylation Assay

As described previously (Wang et al., 2017), methylation assays for human subjects’ DNA extracted from neural tissue were carried out in two independent stages. In the first stage, genome-wide DNA methylation was profiled with DNA of 10 NTD fetuses and eight controls using the Infinium HumanMethylation450 BeadChip (HM450K; Illumina, San Diego, CA, United States). In the second stage, the differentially methylated CpG sites discovered in the first stage were validated in 80 NTD fetuses and 32 controls with the Sequenom MassARRAY system.

QIAamp DNA Mini Kit (QIAGEN, Hilden, Germany) was used for genomic DNA extraction. NanoDrop 2000 Ultramicro spectrophotometer (Thermo Fisher Scientific, MA, United States) was used to quantify and assess the purity of the extracted DNA. Bisulfite conversion was performed with 500 ng DNA utilizing the EZ DNA Methylation Kit (Zymo Research, CA, United States) according to the manufacturer’s protocol. In consideration of conversion efficiency, all samples were prepared in duplicate to curtail potential biases which may induce distorting results. The profile of genome-wide DNA methylation was assessed with the HM450K in accordance with Illumina’s instructions. The methylation status for identified candidate genes was extracted from the array database. In the second stage, validation of the candidate gene identified through microarray assay was conducted with the Sequenom EpiTYPER (Sequenom, San Diego, United States). DNA was amplified with bisulfite-specific PCR. Bisulfite-specific primers were designed with EpiDesigner^1^ as shown in Supplementary Table 1. PCR products were treated with shrimp alkaline phosphatase to dephosphorylate unincorporated dNTP before reverse transcription and base-specific cleavage. After that, the methylation status for each CpG locus was analyzed with the Sequenom mass spectrometer.

The methylation level of the candidate gene in mice was examined with the Sequenom MassARRAY EpiTYPER using DNA isolated from embryo neural tissue. Bisulfite-specific primers were designed using EpiDesigner for amplicon covering the promoter region and 5′ UTR matched with sequence identified in the human candidate gene. The sequence of the primer is listed in the Supplementary Material (Supplementary Table 2).

### Candidate Gene Identification

Candidate genes were identified with hypomethylated NTD-related gene families, using the HM450K array data. Genes with a significantly lower methylation level in cases than in controls were identified using the criteria of *p* < 0.05 and Δβ < −0.2. Based on genes summarized by Copp et al. (2003), Copp and Greene (2010) as a reference, genes and gene families were identified if they had ever been reported to be involved in any disturbance of the cytoskeleton, cell proliferation and neuronal differentiation, neuroepithelial cell death, transcriptional regulation, and chromatin dynamics or sonic hedgehog signaling pathway. Then the identified genes were selected with the following criteria: no significantly hypermethylated CpG site in the promoter region and 5′ UTR and two or more hypomethylated CpG sites in the promoter region and 5′ UTR. Qualified genes with the most hypomethylated CpG sites were chosen as candidate genes for further study. In this study, coordinates were converted from the hg19 genome assembly to GRCh37. The promoter region is defined as within 1,500 bp upstream of the transcription initiation site. TSS200 refers to the 0–200 bases upstream of the transcriptional start site (TSS). TSS1500 is the 200–1500 bases upstream of the TSS, and 5′ UTR is within the 5′ untranslated region, between the TSS and the ATG start site.

### PAH Analysis

As described previously (Wang et al., 2015), PAHs in fetal liver tissues were quantified by gas chromatograph–mass spectrometer. The PAH concentrations were calculated by subtracting the blanks from the detected concentration in the samples and are reported as ng/g lipid. The sum of high-molecular-weight PAHs (H_PAHs), i.e., PAHs with four or five fused aromatic rings, was utilized to represent fetal exposure levels. A total of 53 fetuses were available for PAH analysis in the present study.

### OS Marker Detection

Selected OS markers in fetal neural tissues were detected and quantified by a commercial kit (Nanjing Jiancheng Bioengineering Institute, Nanjing, China). Concentrations of glutathione peroxidase (GPx), superoxide dismutase (SOD), and total antioxidant capacity (TAC) are used as antioxidant indicators. Oxidative macromolecular damage markers included malondialdehyde (MDA) and protein carbonyl (PC), which, respectively, represent lipids and protein oxidation. A total of 20 NTD cases were available for OS analysis in the present study.

### RNA Isolation and Real-Time PCR

Mouse neural tissues were collected, and RNA was extracted with TRIzol (Invitrogen). DNase I digestion (DNA-free, Ambion) was used to remove genomic DNA. Reverse transcription of RNA was conducted immediately with random hexamers (Superscript VILO cDNA synthesis kit). The 7500 Fast Real-Time PCR system (Bio-Rad) was used for performing real-time PCR with iTaq^TM^ Universal SYBR Green Supermix (Bio-Rad). The threshold cycles were calculated with the CFX manager software of Bio-Rad. The primers are given in Supplementary Table 3. *Gapdh* was used to normalize the *Zic4* level.

### Whole-Mount *in situ* Hybridization Assay

To capture the level of candidate gene expression during neural tube closure with lower background and stronger signals, E9.5 mouse embryos were used for whole-mount *in situ* hybridization according to previously described procedures (Huang et al., 2019b). Briefly, digoxigenin-labeled RNA probes for *in situ* hybridizations were synthesized with pGEM-T (Promega) followed by *in vitro* transcription utilizing T7 RNA polymerase (Roche). Mouse embryos were rehydrated and bleached with hydrogen peroxide, followed by incubating with hybridization mix containing digoxigenin-labeled RNA probe. TBST containing 1% sheep serum was used to block the samples at the end of hybridization. Embryos were incubated in developing solution (NBT/BCIP, Roche) for color detection, after which a dissecting microscope was used periodically for photographing.

### Western Blot

Protein extraction was conducted with E10.5 whole embryo using RIPA buffer on ice. The concentration of the extracts was determined with the Bradford assay. Traditional methods were used to perform western blotting. The primary antibodies were rabbit anti-*ZIC4* (1:500, Proteintech, Chicago, United States) and anti-*β*-TUBULIN (1:10,000, Proteintech, Chicago, United States). ImageJ was used to make densitometric comparisons. *β*-Tubulin density measurements were used as loading controls.

### Statistical Analysis

In the human subject study, the χ^2^ test or Fisher’s exact test was used to assess differences in proportions of demographic characteristics between the groups of NTDs and controls. For the HM450K array data, an independent *t*-test was performed in analyses of differentially methylated CpG locus within each gene between cases and controls. Multiple comparison correction was carried out with the Benjamini–Hochberg false discovery rate (FDR) method. CpG loci were determined as significantly hypomethylated in the discovery stage if the following criteria were met: FDR *p* < 0.05 and absolute β difference > 0.2. Shapiro–Wilk test was performed to test the normality of methylation data of *ZIC4* in the validation stage. *t*-Test was conducted to examine the differences in methylation intensity of *ZIC4* between NTD cases and controls validated with the Sequenom MassARRAY system in the validation stage. The association of the *ZIC4* methylation status with risk for NTDs was evaluated by odds ratios (ORs) and 95% confidence intervals (CIs) obtained with logistic regression. In order to acquire a comprehensible interpretation of the coefficient, β-score with negative centuplicate transformation was applied in the logistic model. Maternal occupation and educational level, unplanned pregnancy, parity, and folic acid supplementation were adjusted for in logistic regression. A linear regression was modeled to evaluate the effect of fetal liver H_PAH levels on methylation status of *ZIC4*. Spearman’s correlation analysis was used for exploring the correlation between *ZIC4* methylation and OS markers in neural tissues of NTD fetuses. Data of differentially methylated CpG loci for the *Zic4* gene and the abundance of mRNA and protein are expressed as means ± SEs (or SDs) among embryos from the DMSO, BaP, and BaP and NAC co-treatment groups in the mouse study. One-way analysis of variance (ANOVA) was performed to examine the differences among groups. In all tests, a two-tailed *p* value < 0.05 was considered statistically significant. The statistical package of SPSS 23.0 (IBM Co., Armonk, NY, United States) was used in data analyses.

## Results

### Identification of Candidate Genes

Following our prespecified selection criteria, data analysis of the HM450K assay revealed that *ZIC4*, *CASP8*, *RAB32*, *RARA*, and *TRAF6* were the five most common genes with more hypomethylated CpG sites located within the promoter region and 5′ UTR in NTD cases than in controls (Supplementary Material, Supplementary Table 4). *ZIC4* had 11 significantly different methylation CpG sites in its promoter region and 5′ UTR, with the absolute value of the β difference being greater than 0.2 after Benjamini–Hochberg correction, which ranked first among all reported NTD-related genes in our microarray data.

### Methylation of *ZIC4* Gene in the Microarray Data

According to the HM450K microarray data, 74 probes covered the *ZIC4* gene in the chip, and 22 probes were in the promoter region and 5′ UTR. Forty-nine out of the 74 (66.2%) CpG sites of the *ZIC4* in NTD cases were found to be hypomethylated compared to those in controls. And 13 of the 49 CpG sites were significantly hypomethylated in the promoter region and 5′ UTR. Among those 13 CpG probes, nine (cg03355998, cg21127068, cg06369327, cg05548555, cg26791399, cg15287443, cg26224785, cg06166523, and cg02820514) were located in the TSS1500, and four (cg23957311, cg21639713, cg24620761, and cg02387803) were in the 5′ UTR. In the promoter region and 5′ UTR, 11 out of the 13 hypomethylated CpG sites got Δβ < −0.2, and no hypermethylated CpG site was found. Details of the CpG sites in *ZIC4* are presented in Supplementary Table 5. The schematic diagram for the location of CpG sites in the *ZIC4* gene examined by Infinium HumanMethylation450 BeadChip is presented in Supplementary Figure 10.

### Validation of *ZIC4* Methylation With a Larger Case–Control Cohort

To verify the hypomethylated CpG sites in *ZIC4* that were identified in the above microarray data, the methylation status of CpG sites in the *ZIC4* promoter region and 5′ UTR was assayed with the Sequenom EpiTYPER platform with DNA extracted from the neural tissues of subjects consisting of 80 NTD cases and 32 controls. The demographic characteristics of both mothers and fetuses are shown in the Supplementary Material (Supplementary Table 6).

**FIGURE 1:**
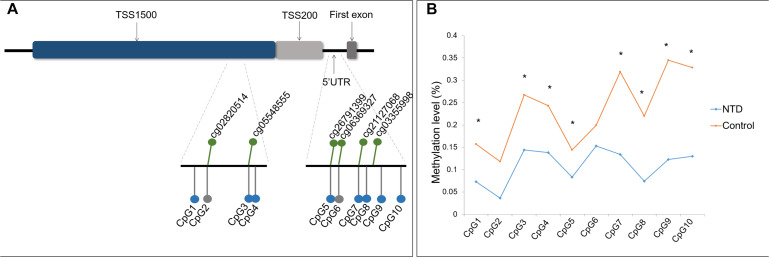
Location and methylation statuses of CpG units examined in *ZIC4*. **(A)** Schematic diagram of CpG sites examined in the *ZIC4* gene. Green dots represent significantly hypomethylated CpG sites discovered by microarray. CpG sites validated with the Sequenom MassARRAY system are indicated as gray (without a significant difference) or blue (significantly hypomethylated) dots. **(B)** Methylation levels of *ZIC4* detected in an independent sample of NTD cases and controls using the Sequenom MassARRAY system. *Difference is significant between NTD cases and non-malformed controls (*p* < 0.05).

Two DNA amplicons targeting the promoter region and 5′ UTR of *ZIC4* were designed to verify the hypomethylated CpG sites identified by microarray analysis (Figure 1A). Amplicon within the TSS1500 region covered four CpG sites (CpG1 to CpG4), among which CpG2 and CpG3 were identical to cg02820514 and cg05548555 discovered in the microarray, respectively. Another amplicon enclosed six CpG sites located in the 5′ UTR (CpG5 to CpG10), in which CpG5, CpG6, and CpG7 were consistent with the cg26791399, cg06369327, and cg21127068 CpG sites, respectively, which were found to be significantly hypomethylated in microarray analysis.

Of these 10 CpG sites, eight demonstrated significant hypomethylation in NTD cases, except for two microarray-matched CpG sites (Figure 1B). All eight CpG sites were kept differentially methylated after applying the Benjamini–Hochberg correction. The β difference of cases and controls was between 6.1 and 22.2%. Detailed information is presented in Supplementary Table 7 of the Supplementary Material.

The average methylation level of *ZIC4* (11.1%) decreased in cases than in controls (24.0%). We utilized logistic regression model to test the association of the mean methylation level of *ZIC4* with the NTD risk. Hypomethylation of *ZIC4* was associated with a higher risk for NTDs (aOR = 1.08; 95% CI: 1.03, 1.13). Increased risk for spina bifida and anencephaly, two major subtypes of NTDs, was also in association with *ZIC4* hypomethylation (Table 1).

**TABLE 1:** Associations between *ZIC4* average methylation and NTD risk in the validation stage.

Group	*n*	Mean methylation (SE)	*p* value	OR (95% CI)	Adjusted OR (95% CI)*^*a*^*
Total NTDs*^*b*^*	80	0.11 (0.07)	<0.001	1.08 (1.04, 1.12)	1.08 (1.03, 1.13)
Spina bifida	39	0.11 (0.03)	<0.001	1.07 (1.03, 1.12)	1.07 (1.01, 1.12)
Anencephaly	33	0.12 (0.06)	<0.001	1.07 (1.02, 1.12)	1.08 (1.01, 1.16)
Controls	32	0.24 (0.19)	–	1.0	1.0

*NTDs, neural tube defects; SE, standard error; OR, odds ratio; and CI, confidence interval. ^*a*^Maternal occupation, maternal educational, periconceptional folic acid supplementation, unplanned pregnancy, and parity were adjusted for. ^*b*^We did not examine the association between *ZIC4* methylation and NTD risk in eight encephalocele cases.*

### *ZIC4* Methylation and PAH Exposure

Polycyclic aromatic hydrocarbon exposure is a proposed risk factor for NTDs (Ren et al., 2011; Wang et al., 2015). We used a linear regression model in a subcohort of the NTD group to investigate the relationship between PAH concentration and methylation level of *ZIC4*. Among the 53 NTD cases, the mean *ZIC4* methylation level was negatively associated with H_PAH concentrations in fetal liver (β [95% CI]: -0.047 [−0.093, −0.001], *p* = 0.046), which indicates that when H_PAHs increase by one unit, there will be a 4.7% decrease in the average methylation level of *ZIC4*. H_PAH and *ZIC4* continued to be negatively associated after adjustment for folic acid use, although the β value did not reach statistical significance (β [95% CI]: −0.045 [−0.091, 0.001], *p* = 0.054). As a supplement, we also determined the correlation between H_PAH level and methylation status for each CpG site in *ZIC4*. Pearson correlation analysis revealed a negative correlation between H_PAHs and the four CpG sites (CpG5, CpG7, CpG8, and CpG10) and the mean methylation level of *ZIC4*. The details for the correlation analysis are presented in Supplementary Material, Supplementary Table 8.

### *ZIC4* Methylation and OS Markers in Human NTD Cases

To determine whether OS is correlated with *ZIC4* methylation level in NTD cases, correlation analyses were carried out in a subcohort of the NTD group, using levels of OS markers (SOD, GPx, TAC, MDA, and PC) and the methylation levels of *ZIC4* in fetal neural tissue. The methylation status of six CpG sites (CpG2, CpG3, CpG4, CpG7, CpG8, and CpG10) presented a moderate to strong inverse correlations with the level of GPx, an antioxidant indicator. Three sites (CpG2, CpG7, and CpG8) were negatively correlated with the protein oxidation marker—PC (Table 2).

**TABLE 2:** Correlation analysis of *ZIC4* methylation statuses and levels of OS markers in neural tissues of NTD cases.

CpG sites	N	SOD		GPx		TAC		MDA		PC	
						
		*ρ*	*p*	*ρ*	*p*	*ρ*	*p*	*ρ*	*p*	*ρ*	*p*
*ZIC4*_1	20	−0.134	0.573	−0.434	0.056	0.069	0.774	−0.155	0.513	−0.257	0.274
*ZIC4*_2	16	−0.123	0.649	−0.712**	0.002	−0.068	0.801	0.190	0.480	−0.748**	0.001
*ZIC4*_3	20	−0.013	0.957	−0.495*	0.027	−0.085	0.721	−0.126	0.597	−0.226	0.338
*ZIC4*_4	20	−0.080	0.736	−0.588**	0.006	0.089	0.710	0.056	0.814	−0.379	0.100
*ZIC4*_5	20	0.122	0.609	−0.299	0.200	0.119	0.618	0.175	0.459	−0.387	0.092
*ZIC4*_6	20	−0.107	0.653	0.130	0.584	0.169	0.476	0.062	0.796	−0.133	0.575
*ZIC4*_7	20	0.046	0.847	−0.465*	0.039	0.140	0.557	0.146	0.538	−0.447*	0.048
*ZIC4*_8	20	−0.141	0.554	−0.469*	0.037	0.026	0.912	0.219	0.354	−0.488*	0.029
*ZIC4*_9	20	0.156	0.511	−0.213	0.368	0.117	0.624	−0.116	0.626	−0.125	0.599
*ZIC4*_10	20	−0.114	0.632	−0.586**	0.007	0.149	0.531	0.267	0.254	−0.399	0.081
*ZIC4*_average	20	0.061	0.799	−0.297	0.203	0.189	0.425	0.002	0.992	−0.342	0.140

OS markers were expressed in unit/mg protein. SOD, superoxide dismutase; GPx, glutathione peroxidase; TAC, total antioxidant capacity; MDA, malondialdehyde; PC, protein carbonyl; and *ρ*, Pearson’s correlation coefficient. Bold values denote statistical significance at the *p* < 0.05 level. *p < 0.05 and **p < 0.01.

### *Zic4* Methylation and Expression in Mouse Embryos Exposed to BaP

A mouse whole-embryo *in vitro* culture model treated with BaP, a well-studied H_PAH, was used to examine the role of PAHs on the *Zic4* methylation indicated in the human study. We previously reported a significantly higher rate of NTD incidence (13%) in BaP-treated mouse embryos (Huang et al., 2019b). In this study, methylation status of an amplicon within the *Zic4* promoter region and 5′ UTR was examined, using DNA extracted from the neural tissue of embryos cultured *in vitro* for 48 h.

**FIGURE 2:**
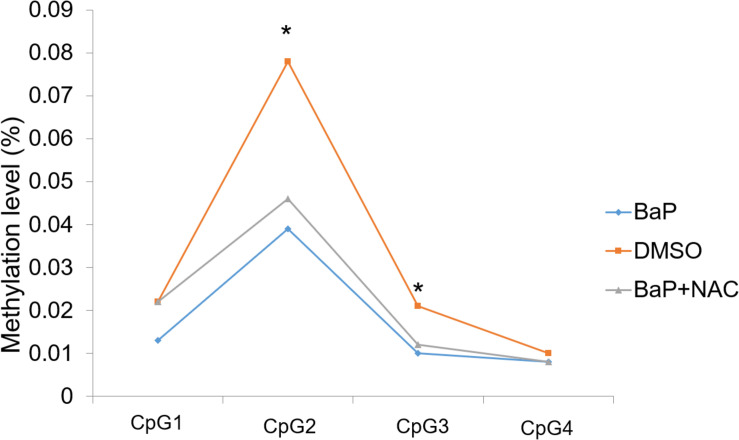
Methylation status of *Zic4* in mouse embryos. DNA isolated from neural tissue of E10.5 mouse in the DMSO, BaP, and BaP and NAC co-treatment groups was utilized to examine the methylation levels of the CpG site covered in the amplicon in the promoter of the *Zic4* gene. *p < 0.05 between the DMSO and BaP groups.

Two of the four detected CpG sites (CpG2 and CpG3) demonstrated significant hypomethylation in mouse embryos after BaP exposure, compared to the DMSO control, with β differences of 3.9% and 1.1%, respectively, (Figure 2). The other two CpG sites also showed decreased methylation levels in the BaP group, although the differences were not statistically significant. The details on the methylation status of *Zic4* of mouse embryos are presented in the Supplementary Material (Supplementary Table 9).

**FIGURE 3:**
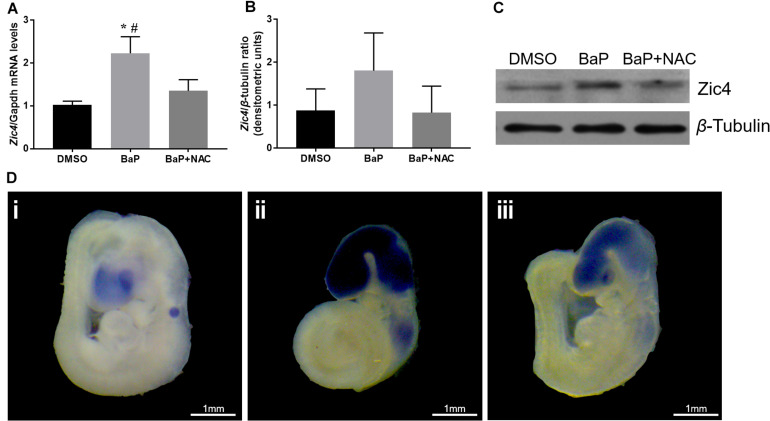
*Zic4* mRNA and protein expressions in mouse embryo after BaP and antioxidant NAC treatments. **(A)** Quantitative real-time PCR analysis of the relative abundance of *Zic4* mRNA normalized to *Gapdh* in neural tissues of embryos treated with BaP or BaP–NAC co-treatment. The transcript levels are means ± SE of two biological replicates (*n* = 5). **(B)** After BaP and BaP–NAC co-treatment, the protein levels of *Zic4* in the whole embryo were measured by western blotting. *β*-Tubulin was used as a loading control, and data are presented as means ± SD (*n* = 5). **(C)** Representative blots of *Zic4* and *β*-tubulin protein levels. **(D)** Representative whole-mount *in situ* hybridizations of *Zic4* expression in E9.5 mouse: (i) DMSO-treated embryos, (ii) BaP-treated embryos, and (iii) BaP- and NAC-co-treated embryos. *p < 0.05 vs. DMSO control; #*p* < 0.05 vs. BaP and NAC co-treatment.

Because decreased methylation in the promoter region and 5′ UTR is expected to upregulate the gene expression, mRNA and protein expression of *Zic4* gene in mouse embryo were examined. According to real-time PCR, *Zic4* mRNA expression was significantly increased in the BaP-treated group compared to the DMSO control group (*p* < 0.05; Figure 3A). Expression of *Zic4* was localized to the neural tissues, especially in the brain and the upper part of the spinal cord, in E9.5 embryos from the BaP group as demonstrated by using *in situ* hybridization (Figure 3D). Western blotting revealed that *Zic4* protein expression level was greater in embryos from the BaP group compared to the control group, although the difference was not statistically significant (Figures 3B,C).

### Effects of Antioxidant on *Zic4* Methylation and Expression

We evaluated whether OS plays a role in *Zic4* methylation during NTD formation. To this end, the methylation status in the promoter region and 5′ UTR of *Zic4* was evaluated using the neural tissue of E10.5 embryos cultured with BaP and antioxidant NAC co-treatment. BaP-induced NTD is rescued by NAC *in vitro* (Huang et al., 2019b). In this study, three of the four detected CpG sites had increased methylation levels in embryos co-treated with BaP and NAC (Figure 2), although the differences were not statistically significant. The details on methylation features of locus-specific CpG sites in *Zic4* are reported in Supplementary Table 9.

The relative abundance of Zic4 mRNA and protein was also examined in embryos cultured in serum with BaP and NAC. NAC normalized *Zic4* mRNA upregulation induced by BaP exposure (*p* < 0.05; Figures 3A,D). Results from *in situ* hybridization indicated that the increased *Zic4* expression in the brain and upper spinal cord was alleviated by co-exposure to NAC. NAC treatment also tended to mitigate elevated protein expression caused by BaP exposure (Figures 3B,C).

## Discussion

Polycyclic aromatic hydrocarbon exposure, which is associated with higher NTD risk, has been reported to cause a reduction in DNA methylation levels in humans (Herbstman et al., 2012; Lee et al., 2017). Increasing evidence suggests a link between DNA methylation dysregulation and NTDs, but to date, no study has identified hypomethylated genes based on a genome-wide methylation profile. Using HM450K microarray data, we identified several hypomethylated genes *ZIC4*, *CASP8*, RAB3*2*, *RARA*, and *TRAF6*. Among these, *CASP8*, *RARA*, and *TRAF6* have been reported to be involved in the NTD formation, and *ZIC4* and *RAB32* are family members of NTD-related genes. Caspase-8, products of *CASP8*, plays the role of initiator in the death receptor-induced apoptotic pathways (Zhao et al., 2009). As a member of the RAR gene family, *RARA* encoded retinoic acid receptor alpha which regulates differentiation, apoptosis, and transcription of clock genes and is essential for normal embryonic development (Elmazar et al., 1997). Also, the protein encoded by *TRAF6* mediates the downstream signal transduction of various cell surface receptors and is reported to play a crucial role in apoptosis during neural tube closure (Lomaga et al., 2000). In sum, *CASP8*, *RARA*, and *TRAF6* may be involved in NTD formation through dysregulation of cell differentiation or apoptosis according to existing studies. *RAB32* belongs to the Rab family that regulates membrane trafficking. Only *RAB23*, a member of the Rab family of small GTPases, has been reported to be associated with NTD development (Hor et al., 2018), while no study on *Rab32* and neural tube closure have been retrieved.

We further validated *ZIC4*, one of the identified genes with most of the hypomethylated CpG sites, using a larger sample of NTD fetuses and non-malformed controls. The mean methylation levels of the detected CpG locus in the promoter region and 5′ UTR of *ZIC4* was negatively associated with H_PAH concentrations in liver tissues of cases that were available for OS analysis in the present study. Likewise, *ZIC4* methylation levels were inversely correlated with OS markers in neural tissues of NTD cases. We performed a further validation utilizing a mouse model in which decreased methylation and elevated expression of *Zic4* was found in embryos from the BaP treatment group in which an increased rate of NTDs has been reported (Huang et al., 2019b). Antioxidant NAC mitigated the *Zic* hypomethylation and upregulation induced by BaP exposure in mouse embryos cultured *in vitro*.

*ZIC4*, the gene with the most hypomethylated CpG sites among qualified genes in the discovery stage, belongs to the *Zic* gene family, which encodes zinc finger proteins. After validating the CpG sites within the *ZIC4* promoter region and 5′ UTR in another independent cohort of subjects, *ZIC4* hypomethylation was found among NTD cases, and a statistically significant association was found between *ZIC4* hypomethylation and increased NTD risk. Zinc finger proteins may interact with key transcriptional coactivators in SHH, WNT, and NODAL signaling pathways, which are known pathways in neural tube closure (Diamand et al., 2018). A study on zebrafish provided evidence that *Zic1* and *Zic4* are crucial for the formation of the dorsal roof plate and dorsal hindbrain (Elsen et al., 2008). In both humans and mice, however, *Zic4* only hampers cerebellum development when there is a combined *trans*-heterozygous loss of *Zic1* and *Zic4* (Diamand et al., 2018). *Zic4* overexpression could inhibit the activation of β-catenin-mediated reporter (Fujimi et al., 2012), which may repress the expression of *Pax3* and *Cdx2*, two vital effectors involved in caudal neural tube closure and/or elongation (Zhao et al., 2014). In support of this, we observed increased expression of mRNA and protein of *Zic4* in mouse embryos with a high NTD rate (Huang et al., 2019b), accompanied by the hypomethylated *Zic4* promoter region and 5′ UTR in neural tissues after BaP exposure. In Xenopus gastrula embryos, a Wnt/β-catenin signaling reporter vector (TOPFLASH vector) was found significantly lowered after coninjection of Zic4 RNA (Fujimi et al., 2012). Overexpression of *Zic4* may hinder the proliferation and specification of neural stem cells via disturbance in Wnt/β-catenin signaling, which could eventually impair the axis formation and the development of the forebrain, midbrain, and hindbrain. Thus, we speculate that *Zic4* hypomethylation might impair neural tube closure through Wnt/β-catenin signaling. In sum, *Zic4* hypomethylation may play a vital role in PAH-induced NTDs, while whether dysregulated *Zic4* expression directly leads to morphological change needs to be further examined in future studies. To the best of our knowledge, no previous study has reported dysregulated methylation of *Zic4* in NTD formation.

PAH exposure is associated with elevated risk for NTD (Ren et al., 2011), and DNA methylation biologically mediates the effects of PAH exposure by affecting the epigenome (Martin and Fry, 2018). Accordingly, we examined the relationship of H_PAH concentrations in fetal liver tissue and the methylation status of the *Zic4* promoter region and 5′ UTR in NTD cases. A linear regression model revealed that hypomethylation of *ZIC4* is associated with H_PAH levels in NTD cases. To further explore the impact of PAH exposure on DNA methylation, we assessed the *Zic4* methylation status using the neural tissue dissected from mouse embryos after BaP exposure, which has also been reported to have an elevated incidence of NTD (Huang et al., 2019b). Levels of locus-specific methylation for two out of the four analyzed CpG sites were significantly hypomethylated in the promoter region and 5′ UTR of *Zic4* after BaP treatment *in vitro*. Although growing evidence suggests that PAH exposure may be correlated with decreased global DNA methylation (Herbstman et al., 2012; Martin and Fry, 2018), the effects of environmental exposure on *Zic4* methylation either *in vivo* or *in vitro* were unknown. Several studies have examined the correlation between PAH concentrations and methylation levels for some target genes, but the inconsistency from studies on humans, animals, and cells suggests that we should interpret our findings with caution. For example, PAH concentrations in maternal serum have been reported to positively correlate with DNA methylation levels of several NTD-related genes, including *CTNNA1*, *PAX3*, and *MYH2* (Wang et al., 2017; Lin et al., 2019); a pilot cross-sectional study conducted in Mexico found that urinary 1-hydroxypyrene, a PAH metabolite, was negatively associated with the methylation levels of interleukin 12 and *p53* gene promoters (Alegria-Torres et al., 2013). These results indicate that DNA methylation regulation may be mediated by PAH exposure, but it remains a challenge to reveal the underlying perplexing mechanisms when it comes to different effects of PAH exposure on distinct genes. Further investigations are required to confirm whether PAH exposure could alter the methylation status of *Zic* genes.

Growing evidence suggests that OS induced by environmental insults may cause DNA methylation dysregulation. To examine the possible mechanisms through which PAH exposure relates to aberrant DNA methylation, we investigated the relationship between OS markers and methylation level of *ZIC4* in fetuses from the NTD group. Several CpG sites in the *ZIC4* promoter region and 5′ UTR were inversely correlated with antioxidant indicators and protein oxidation markers. A whole-embryo culture model was utilized to further explore the role that OS played in DNA methylation aberration after BaP exposure. We previously showed that NAC could rescue NTDs and alleviate OS induced by BaP exposure *in vitro* (Huang et al., 2019b). After detecting the *Zic4* methylation level in mouse embryos developed in culture media with BaP and BaP–NAC co-treatment, we found that NAC treatment partially attenuated the hypomethylation of *Zic4* and normalized upregulated gene expression in neural tissues. NAC is a by-product of glutathione and is essential in the maintenance and metabolism of glutathione, which can prevent OS and its negative downstream effects (Kerksick and Willoughby, 2005). NAC treatment rescues global hypomethylation induced by OS after arsenic exposure, in both human THP-1 cells (Song et al., 2019) and chicken embryos (Han et al., 2011). In addition, according to a Vienna randomized trial which aimed to explore the impact of antioxidant-rich diet on the epigenetic profile of genes encoding mismatch repair enzymes, the methylation status of MLH1 is negatively correlated with DNA strand breaks induced by OS, and there is an increase in MLH1 methylation status after antioxidant intervention. This study indicated that antioxidants might lead to an increase in methylation status (Switzeny et al., 2012). All of this evidence consistently supports our hypothesis that OS mediates methylation dysregulation after PAH exposure. It has been reported that concentrations of 8-hydroxy-2′-deoxyguanosine (8-OHdG), an informative biomarker of DNA oxidative damage, are significantly increased in the maternal serum of NTD cases compared to that of controls (Yuan et al., 2015). Evidence from human cell and mouse studies indicate that 8-OHdG may inhibit DNA methylation by suppressing human DNA methyltransferase (DNMT) and murine Dnmt3a (Turk et al., 1995; Maltseva et al., 2009). Moreover, OS may induce excessive *S*-adenosyl-L-methionine consumption which would cause a decrease in methyl donor and then block methylation reaction. Furthermore, the upregulation of the demethylation enzyme ten-eleven translocation may also play a role in OS-induced hypomethylation (Chia et al., 2011; Huang et al., 2019a). Although no other specific evidence for *Zic4* methylation and OS is available in the literature, the negative correlation between markers of OS and *ZIC4* methylation in fetuses, together with hypomethylated *Zic*4 after BaP exposure rescued by NAC in mouse embryos, suggests that PAHs may mediate *ZIC4*/*Zic4* methylation via OS.

A strength of this study is that we used neural tissues to detect tissue-specific DNA methylation, which is crucial for elucidating the etiology of NTD. However, our results should be considered alongside the limitations mentioned below. We did not investigate transcript-expression data for *ZIC4* in fetuses because it can be quite challenging to collect tissues from terminated NTD cases that are fresh enough for RNA assays. However, the mouse model may provide some clues for *Zic4* expression as compensation. In addition, we only detected methylation levels within the promoter region and 5′ UTR because that is the most studied region. Future studies should take methylation status in other regulatory regions into consideration.

## Conclusion

Our study is the first to show that hypomethylated *ZIC4* in neural tissues is associated with an elevated NTD risk. The findings from the human study and the mouse model provide novel evidence for the assumption that OS is a possible mechanism contributing to the methylation change of *ZIC4*/*Zic4* in response to PAH exposure in NTD formation.

## Data Availability Statement

The raw data supporting the conclusions of this article will be made available by the authors, without undue reservation.

## Ethics Statement

The studies involving human participants were reviewed and approved by Institutional Review Board of Peking University. Written informed consent to participate in this study was provided by the participants’ legal guardian/next of kin. The animal study was reviewed and approved by The Institutional Animal Care and Use Committee at Peking University.

## Author Contributions

YH conducted the experiments, analyzed the data, and drafted the manuscript. SL contributed to the study design and animal experiments. CW provided critical comments on the draft manuscript. XP contributed to the animal experiments and data analyses. LJ and ZL contributed to study design and participant recruitment. AR and LW conceptualized the study, supervised the project, and critically revised the manuscript. All authors read and approved the final manuscript.

## Conflict of Interest

The authors declare that the research was conducted in the absence of any commercial or financial relationships that could be construed as a potential conflict of interest.

## Footnotes

1 http://www.epidesigner.com/

## Abbreviations

PAHs: polycyclic aromatic hydrocarbons
NTDs: neural tube defects
*ZIC4/Zic4*: Zic family member 4
BaP: benz(a)pyrene
OS: oxidative stress
NAC: *N*-acetyl -L-cysteine
E: embryonic day
HM450K: Infinium HumanMethylation450 BeadChip
TAC: total antioxidant capacity
SOD: superoxide dismutase
GPx: glutathione peroxidase
PC: protein carbonyl
MDA: malondialdehyde
H_PAHs: high-molecular-weight PAHs
FDR: false discovery rate
DNMT: DNA methyltransferase
SAM: *S*-adenosyl -L-methionine.
