# Supplementary material for: Neural Tube Defects and ZIC4 Hypomethylation in Relation to Polycyclic Aromatic Hydrocarbon Exposure
Source: Front Cell Dev Biol. 2020 Nov 16;8:582661. doi: 10.3389/fcell.2020.582661 (PMC7701213; doi:10.3389/fcell.2020.582661)
Supplement: Supplementary file 1 [file Data_Sheet_1.docx]

**Supplementary Material**

Neural tube defects and *ZIC4* hypomethylation in relation to polycyclic aromatic hydrocarbon exposure: A case-control study

Yun Huang, Shanshan Lin, Chengrong Wang, Xin Pi, Lei Jin, Zhiwen Li, Linlin Wang*, Aiguo Ren*

**Table of Contents**

**S1 Table.** The PCR primer sequences in Sequenom EpiTYPER sequencing for human.

**S2 Table.** The PCR primer sequences in Sequenom EpiTYPER sequencing for mouse.

**S3 Table.** The sequences of primer for real-time PCR.

**S4 Table** Top 5 genes with multiple hypomethylated CpG sites^a^ between the NTD cases and controls.

**S5 Table.** Methylation of *ZIC4* gene using the Human Methylation 450 Bead Chip assay.

**S6 Table.** Demographic and obstetric characteristics of NTD cases and controls in Shanxi Province, China, 2011–2014.

**S7 Table.** Validation of differentially methylated CpG sites of *ZIC4* gene in neural tissues of NTD cases and controls with Sequenom EpiTYPER.

**S8 Table.** Correlation analysis of differentially methylated CpG sites and PAH concentrations in maternal serum in NTD cases.

**S9 Table.** Differentially methylated CpG sites in *Zic4* in neural tissues of mice embryo with and without BaP exposure/NAC rescue.

**S10 Figure.** Location of CpG sites in the ZIC4 gene examined by Infinium HumanMethylation450 BeadChip.

## **S1 Table.** The PCR primer sequences in Sequenom EpiTYPER sequencing for human.

| Primer | Sequence |
| --- | --- |
| Amplicon_1F | aggaagagagTTATTAATTTTGAGTGGAATGGAATG |
| Amplicon_1R | cagtaatacgactcactatagggagaaggctCCCTTAAATCCCCTCCCTTATTAT |
| Amplicon2_F | aggaagagagATAATAAGGGAGGGGATTTAAGGG |
| Amplicon2_R | cagtaatacgactcactatagggagaaggctCAAACCTCTCTTTTATAATTCCAAC |

## **S2 Table.** The PCR primer sequences in Sequenom EpiTYPER sequencing for mouse.

| Primer | Sequence |
| --- | --- |
| Amplicon_1F | aggaagagagAAAGTTTGTTTGTTTATTTTGAGGAGA |
| Amplicon_1R | cagtaatacgactcactatagggagaaggctAAAACCTTTTCTAAACCCAAACTC |

## **S3 Table.** The sequences of primer for real-time PCR.

| Primer | Sequence |
| --- | --- |
| *Zic4*_F | TCTGGCTACGACTCGGCTAT |
| *Zic4*_R | CATCATTCGCTCACGTCTGT |
| *Gapdh*_F | ATGACATCAAGAAGGTGGTG |
| *Gapdh*_R | CATACCAGGAAATGAGCTTG |

## **S4 Table** Top 5 genes with multiple hypomethylated CpG sites^a^ between the NTD cases and controls.

| Gene | Illumina ID | *β*-diff | Ad-*p* | Region |
| --- | --- | --- | --- | --- |
| *ZIC4* (11 sites) | cg02820514 | -0.215 | 0.030207 | TSS1500 |
|  | cg03355998 | -0.267 | 0.010098 | TSS1500 |
|  | cg05548555 | -0.228 | 0.02452 | TSS1500 |
|  | cg06369327 | -0.239 | 0.027849 | TSS1500 |
|  | cg15287443 | -0.213 | 0.00609 | TSS1500 |
|  | cg21127068 | -0.302 | 0.01316 | TSS1500 |
|  | cg21639713 | -0.206 | 0.038019 | 5'UTR |
|  | cg24620761 | -0.223 | 0.048069 | 5'UTR |
|  | cg26224785 | -0.290 | 0.001633 | TSS1500 |
|  | cg02387803 | -0.274 | 0.040587 | 5'UTR |
|  | cg26791399 | -0.302 | 0.023553 | TSS1500 |
| *CASP8* (9 sites) | cg04048517 | -0.251 | 0.000174 | TSS1500 |
|  | cg09464206 | -0.294 | 0.002164 | 5'UTR |
|  | cg00978584 | -0.399 | 1.14E-05 | 5'UTR |
|  | cg04286206 | -0.217 | 0.000948 | 5'UTR |
|  | cg14962032 | -0.414 | 5.19E-05 | 5'UTR |
|  | cg20418725 | -0.390 | 0.000487 | 5'UTR |
|  | cg23882545 | -0.476 | 0.000273 | 5'UTR |
|  | cg25748441 | -0.371 | 0.001451 | 5'UTR |
|  | cg27410837 | -0.284 | 0.000504 | 5'UTR |
| *RAB32* (3 sites) | cg01915609 | -0.250 | 0.000162 | TSS1500 |
|  | cg24744430 | -0.264 | 0.000343 | TSS1500 |
|  | cg25634742 | -0.276 | 0.000192 | TSS1500 |
| *RARA* (2 sites) | cg08580254 | -0.250 | 6.18E-06 | 5'UTR |
|  | cg19572487 | -0.317 | 0.002361 | 5'UTR |
| *TRAF6* (2 sites) | cg07170183 | -0.219 | 0.000129 | TSS1500 |
|  | cg23624321 | -0.226 | 0.003306 | TSS1500 |

Note: *β*-diff, *β* difference; Ad-*p*, adjusted *p*-value.

^a^ Selection criteria: (1) the absolute value of mean methylation difference of CpG site between NTD case and control tissues is >20%; (2) adjusted *p*-value < 0.05.

## **S5 Table.** Methylation of *ZIC4* gene using the Human Methylation 450 Bead Chip assay.

| Illumina ID | Chr | Mapinnfo | Case | Control | β-diff | ad-p | region |
| --- | --- | --- | --- | --- | --- | --- | --- |
| cg02820514 | 3 | 147124827 | 0.156015 | 0.371127 | -0.21511 | 0.030207 | TSS1500 |
| cg26791399 | 3 | 147124687 | 0.264833 | 0.566621 | -0.30179 | 0.023553 | TSS1500 |
| cg05548555 | 3 | 147124543 | 0.1132 | 0.341351 | -0.22815 | 0.02452 | TSS1500 |
| cg06369327 | 3 | 147124523 | 0.096978 | 0.335894 | -0.23892 | 0.027849 | TSS1500 |
| cg21127068 | 3 | 147124417 | 0.151442 | 0.453109 | -0.30167 | 0.01316 | TSS1500 |
| cg03355998 | 3 | 147124363 | 0.115771 | 0.382761 | -0.26699 | 0.010098 | TSS1500 |
| cg02387803 | 3 | 147123457 | 0.376503 | 0.65069 | -0.27419 | 0.040587 | 5'UTR |
| cg24620761 | 3 | 147123199 | 0.39232 | 0.615575 | -0.22325 | 0.048069 | 5'UTR |
| cg21639713 | 3 | 147123123 | 0.497728 | 0.703765 | -0.20604 | 0.038019 | 5'UTR |
| cg06166523 | 3 | 147122664 | 0.39853 | 0.593158 | -0.19463 | 0.046711 | TSS1500 |
| cg26224785 | 3 | 147122315 | 0.428968 | 0.719438 | -0.29047 | 0.001633 | TSS1500 |
| cg23957311 | 3 | 147121892 | 0.749283 | 0.906971 | -0.15769 | 0.001511 | 5'UTR |
| cg15287443 | 3 | 147121229 | 0.61449 | 0.827164 | -0.21267 | 0.00609 | TSS1500 |
| cg00896370 | 3 | 147116807 | 0.304774 | 0.714755 | -0.40998 | 0.000873 | Body |
| cg25449440 | 3 | 147116420 | 0.401798 | 0.772792 | -0.37099 | 0.001905 | Body |
| cg03388789 | 3 | 147116135 | 0.279242 | 0.598281 | -0.31904 | 0.005248 | Body |
| cg06070263 | 3 | 147115930 | 0.254754 | 0.410733 | -0.15598 | 0.041571 | Body |
| cg18731327 | 3 | 147114352 | 0.283229 | 0.485455 | -0.20223 | 0.026451 | Body |
| cg12892506 | 3 | 147113918 | 0.096326 | 0.334337 | -0.23801 | 0.019974 | Body |
| cg18082337 | 3 | 147113726 | 0.172749 | 0.485253 | -0.3125 | 0.009734 | Body |
| cg05855917 | 3 | 147113700 | 0.157446 | 0.53145 | -0.374 | 0.001838 | Body |
| cg02390329 | 3 | 147113092 | 0.513861 | 0.840911 | -0.32705 | 0.001525 | Body |
| cg04557018 | 3 | 147112605 | 0.136447 | 0.37697 | -0.24052 | 0.007342 | Body |
| cg18292434 | 3 | 147112541 | 0.248519 | 0.581217 | -0.3327 | 0.001405 | Body |
| cg20939084 | 3 | 147109784 | 0.095275 | 0.361456 | -0.26618 | 0.012101 | Body |
| cg18930354 | 3 | 147109629 | 0.092137 | 0.296143 | -0.20401 | 0.026043 | Body |
| cg17569743 | 3 | 147109490 | 0.197157 | 0.458912 | -0.26175 | 0.009049 | Body |
| cg08393041 | 3 | 147109426 | 0.250416 | 0.629412 | -0.379 | 0.007768 | Body |
| cg16240162 | 3 | 147106890 | 0.351707 | 0.157253 | 0.194454 | 0.041696 | Body |
| cg04079301 | 3 | 147106635 | 0.141011 | 0.05133 | 0.089681 | 0.039076 | Body |
| cg04556126 | 3 | 147106561 | 0.360916 | 0.098131 | 0.262785 | 0.03038 | Body |
| cg08889797 | 3 | 147106489 | 0.332562 | 0.101501 | 0.231061 | 0.022324 | Body |
| cg22614239 | 3 | 147106208 | 0.358973 | 0.143533 | 0.21544 | 0.048756 | Body |
| cg13278496 | 3 | 147125287 | 0.095196 | 0.099206 | -0.00401 | 0.945486 | TSS1500 |
| cg13015925 | 3 | 147125114 | 0.086485 | 0.165949 | -0.07946 | 0.054038 | TSS1500 |
| cg19516404 | 3 | 147123475 | 0.409798 | 0.552027 | -0.14223 | 0.07905 | TSS200 |
| cg16790847 | 3 | 147123429 | 0.428399 | 0.590797 | -0.1624 | 0.117863 | 5'UTR |
| cg03631131 | 3 | 147115506 | 0.102801 | 0.158708 | -0.05591 | 0.220371 | Body |
| cg08091192 | 3 | 147114986 | 0.137184 | 0.163547 | -0.02636 | 0.710406 | Body |
| cg00028935 | 3 | 147114406 | 0.170663 | 0.296078 | -0.12541 | 0.063793 | Body |
| cg02126051 | 3 | 147112438 | 0.104099 | 0.173444 | -0.06934 | 0.069263 | Body |
| cg22203776 | 3 | 147112316 | 0.082753 | 0.109251 | -0.0265 | 0.393975 | Body |
| cg25007283 | 3 | 147112143 | 0.163311 | 0.228058 | -0.06475 | 0.341218 | Body |
| cg26014036 | 3 | 147112096 | 0.077733 | 0.089353 | -0.01162 | 0.76307 | Body |
| cg02653559 | 3 | 147112081 | 0.074203 | 0.078287 | -0.00408 | 0.867465 | Body |
| cg13340636 | 3 | 147110667 | 0.055463 | 0.065893 | -0.01043 | 0.270898 | Body |
| cg01581018 | 3 | 147110595 | 0.050207 | 0.054144 | -0.00394 | 0.771472 | Body |
| cg12388007 | 3 | 147110499 | 0.038007 | 0.039769 | -0.00176 | 0.837972 | Body |
| cg00235367 | 3 | 147110322 | 0.028216 | 0.028813 | -0.0006 | 0.891392 | Body |
| cg15105326 | 3 | 147108916 | 0.466228 | 0.552743 | -0.08652 | 0.372646 | Body |
| cg16768018 | 3 | 147108843 | 0.39033 | 0.525244 | -0.13491 | 0.237426 | Body |
| cg26790247 | 3 | 147108512 | 0.651055 | 0.757655 | -0.1066 | 0.500681 | Body |
| cg23980740 | 3 | 147107900 | 0.430884 | 0.490682 | -0.0598 | 0.701037 | Body |
| cg01137401 | 3 | 147107349 | 0.676041 | 0.736337 | -0.0603 | 0.65888 | Body |
| cg24686610 | 3 | 147125782 | 0.102782 | 0.077132 | 0.025649 | 0.153216 | TSS1500 |
| cg11142389 | 3 | 147125765 | 0.16757 | 0.109495 | 0.058076 | 0.351452 | TSS1500 |
| cg08070771 | 3 | 147125758 | 0.145553 | 0.084368 | 0.061185 | 0.123834 | TSS1500 |
| cg17546247 | 3 | 147125714 | 0.184196 | 0.110697 | 0.073499 | 0.168416 | TSS1500 |
| cg23189410 | 3 | 147125712 | 0.111998 | 0.074157 | 0.037841 | 0.284699 | TSS1500 |
| cg13897134 | 3 | 147120248 | 0.77798 | 0.760349 | 0.017631 | 0.777672 | Body |
| cg08013557 | 3 | 147115043 | 0.14363 | 0.141377 | 0.002253 | 0.971526 | Body |
| cg03900143 | 3 | 147111660 | 0.059951 | 0.047533 | 0.012418 | 0.4975 | Body |
| cg17003736 | 3 | 147111308 | 0.073491 | 0.06261 | 0.01088 | 0.315073 | Body |
| cg00154357 | 3 | 147111280 | 0.086812 | 0.050361 | 0.03645 | 0.321512 | Body |
| cg03881775 | 3 | 147111135 | 0.051937 | 0.042793 | 0.009144 | 0.461881 | Body |
| cg27606499 | 3 | 147111120 | 0.086227 | 0.082046 | 0.004181 | 0.609492 | Body |
| cg07850418 | 3 | 147110378 | 0.027012 | 0.025765 | 0.001246 | 0.806688 | Body |
| cg08812189 | 3 | 147110367 | 0.032265 | 0.031079 | 0.001186 | 0.883209 | Body |
| cg22796509 | 3 | 147110295 | 0.073752 | 0.063977 | 0.009775 | 0.238258 | Body |
| cg27416372 | 3 | 147110229 | 0.04494 | 0.042894 | 0.002046 | 0.588388 | Body |
| cg11139044 | 3 | 147107109 | 0.463479 | 0.430191 | 0.033287 | 0.783336 | Body |
| cg00334063 | 3 | 147106010 | 0.434729 | 0.201976 | 0.232753 | 0.058304 | Body |
| cg12976081 | 3 | 147105899 | 0.424067 | 0.281249 | 0.142817 | 0.124998 | Body |
| cg07406191 | 3 | 147105190 | 0.610195 | 0.448481 | 0.161714 | 0.320703 | Body |

Note: The Illumina 450k methylation chip contains 485,512 individual CpG sites, spread across 99% of RefSeq genes (21,306 protein-coding genes). Illumina ID is identified according to Human Methylation 450 Bead Chip. The nucleotide position is based on NCBI build 37/hg19. Region is defined relative to the nearest open reading frame: within 1500 (TSS1500) or 200 bp (TSS200) of a transcription start site, in the 5’ UTR, the first exon of a transcript (exon) and in the body of gene (body). Differentially methylated CpG sites were identified by two criterions: the false discovery rate < 0.05, which was analyzed by independent t-tests with multiple comparison tests; the absolute β-value difference > 0.05.

Chr, chromosome; Mapinfo, nucleotide position; *β*-diff, difference of *β* value between NTD cases and controls; ad-*p*, adjusted *p*-value; UTR, untranslated region.

## **S6 Table.** Demographic and obstetric characteristics of NTD cases and controls in Shanxi Province, China, 2011–2014.

| Characteristic | Cases  *N* (%) | Controls*^a^*  *N* (%) | *p* value*^b^* |
| --- | --- | --- | --- |
| Maternal age (y) |  |  | 0.563 |
| <25 | 34 (43.6) | 16 (55.2) |  |
| 25-29 | 21 (26.9) | 6 (20.7) |  |
| ≥30 | 23 (29.5) | 7 (24.1) |  |
| BMI (kg/m2) |  |  |  |
| <18.5 | 8 (10.5) | 2 (6.5) | 0.639 |
| 18.5-24.9 | 42 (55.3) | 20 (64.5) |  |
| ≥25 | 26 (34.2) | 9 (29.0) |  |
| Maternal education |  |  | 0.004 |
| Primary or lower | 9 (11.3) | 4 (12.5) |  |
| Junior high | 57 (71.3) | 13 (40.6) |  |
| High school or above | 14 (17.5) | 15 (46.9) |  |
| Occupation |  |  | <0.001 |
| Farmer | 67 (85.9) | 15 (46.9) |  |
| Non-farmer | 11 (14.1) | 17 (53.1) |  |
| Previous birth defects history |  |  | 0.577 |
| Yes | 4 (5.0) | 0 (0.0) |  |
| No | 76 (95.0) | 32 (100.0) |  |
| Gravidity |  |  | 0.146 |
| 1 | 32 (40.5) | 18 (56.3) |  |
| ≥2 | 47 (59.5) | 14 (43.8) |  |
| Parity |  |  | 0.028 |
| 1 | 33 (46.5) | 20 (71.4) |  |
| ≥2 | 38 (53.5) | 8 (28.6) |  |
| Unplanned pregnancy |  |  | 0.001 |
| Yes | 30 (38.0) | 23 (74.2) |  |
| No | 49 (62.0) | 8 (25.8) |  |
| Periconceptional folate supplementation |  |  | 0.001 |
| Yes | 47 (59.5) | 8 (25.0) |  |
| No | 32 (40.5) | 24 (75.0) |  |
| Fever or flu during early pregnancy |  |  | 0.112 |
| Yes | 28 (35.4) | 6 (18.8) |  |
| No | 51 (64.6) | 26 (81.3) |  |
| Active or passive smoking |  |  | 0.296 |
| Yes | 49 (61.3) | 16 (50.0) |  |
| No | 31 (38.8) | 16 (50.0) |  |
| Drinking |  |  | 1.000 |
| Yes | 3 (3.8) | 1 (3.1) |  |
| No | 77 (96.3) | 31 (96.9) |  |
| Primary fuel used for cooking |  |  | 1.000 |
| Coal | 13 (16.5) | 5 (15.6) |  |
| Natural gas/other | 66 (83.5) | 27 (84.4) |  |
| Primary fuel used for heating |  |  | 0.754 |
| Coal | 11 (13.9) | 3 (9.4) |  |
| Natural gas/other | 68 (86.1) | 29 (90.6) |  |
| Gestational age (weeks) |  |  | 0.169 |
| 13-27 | 56 (71.8) | 17 (56.7) |  |
| ≥28 | 22 (28.2) | 13 (43.3) |  |
| Fetus sex |  |  | 0.287 |
| Male | 35 (47.9) | 18 (60.0) |  |
| Female | 38 (52.1) | 12 (40.0) |  |

^a^ The total number may not be equal to the number of cases or controls due to missing or unknown data.

^b^ The χ2 test or Fisher's exact test were used to compare demographic information between the case and control groups.

NTD, neural tube defect.

## **S7 Table.** Validation of differentially methylated CpG sites of *ZIC4* gene in neural tissues of NTD cases and controls with Sequenom EpiTYPER.

| **CpG site** | **Cases** | | |  | **Controls** | | |  | **Difference** | | | | ***p* value** |
| --- | --- | --- | --- | --- | --- | --- | --- | --- | --- | --- | --- | --- | --- |
|  | **N** | **Mean** | **SD** |  | **N** | **Mean** | **SD** |  | **Mean** | **Std. Error** | **95% Confidence Interval** | |  |
| *ZIC4*_1 | 78 | 0.073 | 0.057 |  | 32 | 0.157 | 0.14 |  | 0.084 | 0.026 | 0.032 | 0.136 | 0.002 |
| *ZIC4*_2 | 57 | 0.036 | 0.06 |  | 23 | 0.118 | 0.218 |  | 0.082 | 0.046 | -0.013 | 0.178 | 0.088 |
| *ZIC4*_3 | 77 | 0.144 | 0.081 |  | 32 | 0.267 | 0.201 |  | 0.123 | 0.037 | 0.049 | 0.198 | 0.002 |
| *ZIC4*_4 | 80 | 0.138 | 0.068 |  | 32 | 0.243 | 0.183 |  | 0.105 | 0.033 | 0.037 | 0.172 | 0.003 |
| *ZIC4*_5 | 80 | 0.083 | 0.126 |  | 32 | 0.144 | 0.137 |  | 0.061 | 0.028 | 0.005 | 0.117 | 0.033 |
| *ZIC4*_6 | 80 | 0.153 | 0.179 |  | 31 | 0.199 | 0.134 |  | 0.046 | 0.035 | -0.024 | 0.117 | 0.192 |
| *ZIC4*_7 | 80 | 0.134 | 0.081 |  | 32 | 0.319 | 0.267 |  | 0.185 | 0.048 | 0.087 | 0.283 | 0.001 |
| *ZIC4*_8 | 80 | 0.074 | 0.069 |  | 32 | 0.22 | 0.191 |  | 0.147 | 0.035 | 0.076 | 0.217 | 0.000 |
| *ZIC4*_9 | 80 | 0.123 | 0.111 |  | 32 | 0.345 | 0.301 |  | 0.222 | 0.055 | 0.111 | 0.333 | 0.000 |
| *ZIC4*_10 | 80 | 0.13 | 0.11 |  | 32 | 0.328 | 0.287 |  | 0.198 | 0.052 | 0.092 | 0.304 | 0.001 |
| *ZIC4*_average | 80 | 0.111 | 0.071 |  | 32 | 0.24 | 0.192 |  | 0.129 | 0.025 | 0.08 | 0.178 | 0.000 |

Note: analyzed by independent t-tests. SD, standard deviation; Std Error, standard error.

## **S8 Table.** Correlation analysis of differentially methylated CpG sites and PAH concentrations in maternal serum in NTD cases.

|  |  | Total PAHs, | | L_PAHs, | | H_PAHs, | |
| --- | --- | --- | --- | --- | --- | --- | --- |
|  |  | ng/g lipid | | ng/g lipid | | ng/g lipid | |
|  | N | ρ | ***p*** | ρ | ***p*** | ρ | ***p*** |
| *ZIC4*_1 | 51 | -0.166 | 0.245 | -0.138 | 0.336 | -0.155 | 0.278 |
| *ZIC4*_2 | 36 | -0.237 | 0.165 | -0.234 | 0.170 | -0.265 | 0.119 |
| *ZIC4*_3 | 51 | -0.253 | 0.073 | -0.261 | 0.064 | -0.224 | 0.114 |
| *ZIC4*_4 | 53 | -0.265 | 0.056 | -0.267 | 0.053 | -0.260 | 0.060 |
| *ZIC4*_5 | 53 | -.307^*^ | 0.025 | -.309^*^ | 0.024 | -.295^*^ | 0.032 |
| *ZIC4*_6 | 53 | -0.127 | 0.365 | -0.153 | 0.273 | -0.100 | 0.476 |
| *ZIC4*_7 | 53 | -.280^*^ | 0.042 | -.279^*^ | 0.043 | -.283^*^ | 0.040 |
| *ZIC4*_8 | 53 | -.330^*^ | 0.016 | -.307^*^ | 0.025 | -.347^*^ | 0.011 |
| *ZIC4*_9 | 53 | -0.218 | 0.117 | -0.236 | 0.089 | -0.170 | 0.223 |
| *ZIC4*_10 | 53 | -.276^*^ | 0.045 | -0.240 | 0.083 | -.318^*^ | 0.020 |
| *ZIC4*_average | 53 | -.383^**^ | 0.005 | -.382^**^ | 0.005 | -.339^*^ | 0.013 |

Note: PAHs, polycyclic aromatic hydrocarbons; total PAHs, sum of all PAHs; L_PAHs, sum of low-molecular-weight PAHs, including acenaphthylene, acenaphthene, fluorene, phenanthrene, anthracene, fluoranthene, and retene; H_PAHs, sum of high-molecular-weight PAHs, including pyrene, benz[a]anthracene, chrysene, benzo[b]fluoranthene, benzo[k]fluoranthene, and benzo[a]pyrene; ρ, Pearson’s correlation coefficient.

## **S9 Table.** Differentially methylated CpG sites in *Zic4* in neural tissues of mice embryo with and without BaP exposure/NAC rescue.

| **CpG site** | **DMSO controls (1)** | | |  | **BaP (2)** | | |  | **BaP+NAC (3)** | | | **Difference between (1) and (2)** | **Difference between (2) and (3)** | **Difference between (1) and (3)** |
| --- | --- | --- | --- | --- | --- | --- | --- | --- | --- | --- | --- | --- | --- | --- |
|  | **N** | **Mean** | **SD** |  | **N** | **Mean** | **SD** |  | **N** | **Mean** | **SD** | ***p* value** | ***p* value** | ***p* value** |
| *Zic4*_1 | 6 | 0.022 | 0.017 |  | 11 | 0.013 | 0.017 |  | 10 | 0.022 | 0.026 | 0.407 | 0.32 | 0.976 |
| *Zic4*_2 | 21 | 0.078 | 0.068 |  | 22 | 0.039 | 0.033 |  | 21 | 0.046 | 0.038 | 0.012 | 0.634 | 0.04 |
| *Zic4*_3 | 21 | 0.021 | 0.016 |  | 22 | 0.01 | 0.01 |  | 22 | 0.012 | 0.014 | 0.01 | 0.741 | 0.024 |
| *Zic4*_4 | 20 | 0.01 | 0.011 |  | 22 | 0.008 | 0.01 |  | 22 | 0.008 | 0.014 | 0.631 | 0.899 | 0.721 |

Note: differentially methylated CpG sites of *Zic4* were detected by one-way analysis of variance.


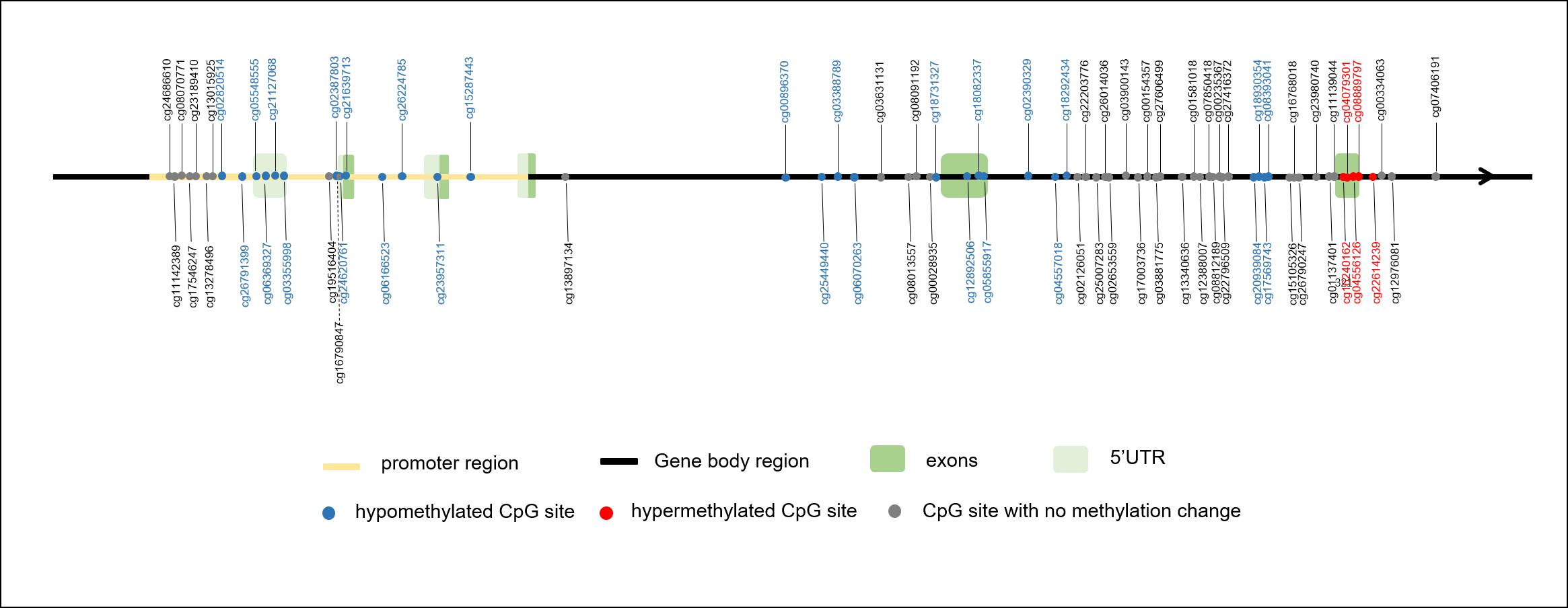


## **S10 Figure.** Location of CpG sites in the ZIC4 gene examined by Infinium HumanMethylation450 BeadChip.

The promoter region is defined as within 1500 bp upstream of the transcription initiation site. Gene body is defined as the entire gene from the exons to the end of the transcript. The black arrow indicates the direction of the sequence. The coding exons are rendered in dark green, and the promoter (i.e. TSS200, TSS1500) and 5'UTR regions are rendered in a lighter shade of green.
