# Supplementary figures and images for: Neural Tube Defects and ZIC4 Hypomethylation in Relation to Polycyclic Aromatic Hydrocarbon Exposure
Source: Front Cell Dev Biol. 2020 Nov 16;8:582661. doi: 10.3389/fcell.2020.582661 (PMC7701213; doi:10.3389/fcell.2020.582661)

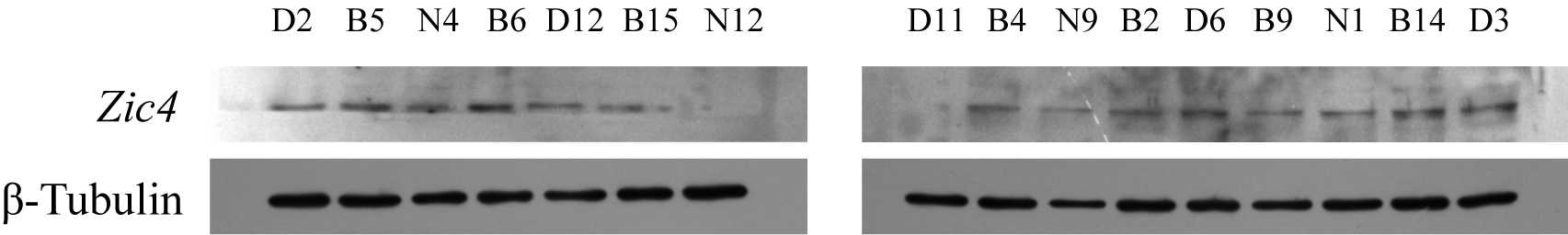

Supplement: Supplementary file 2 [file Image_1.TIF]

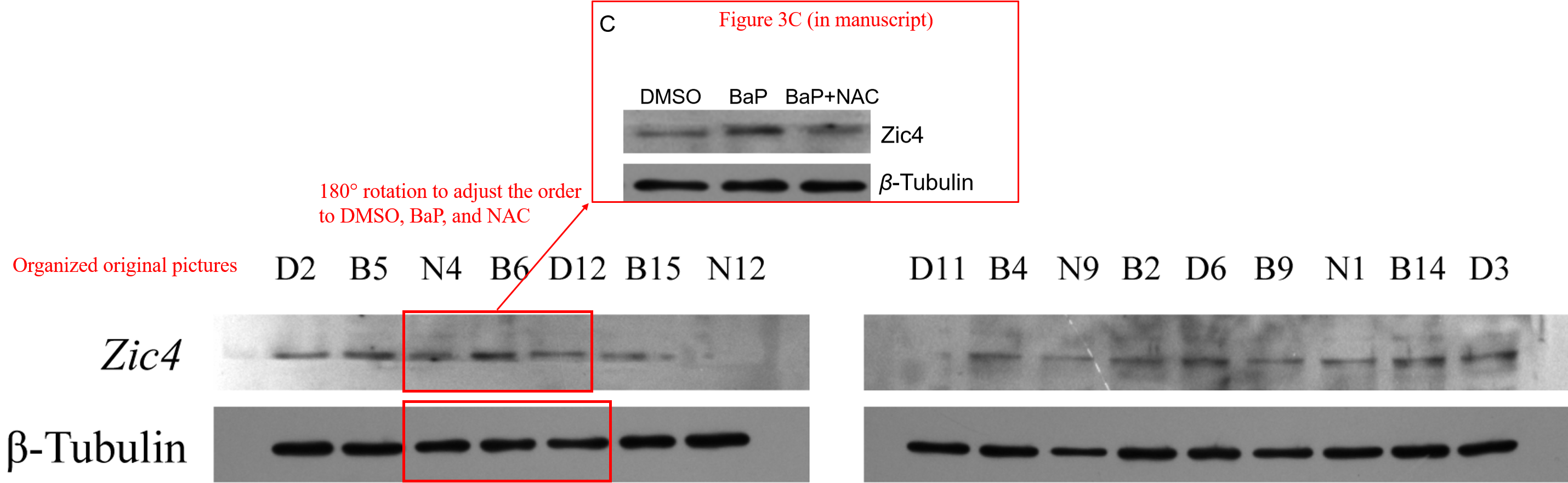

Supplement: Supplementary file 3 [file Image_2.PNG]

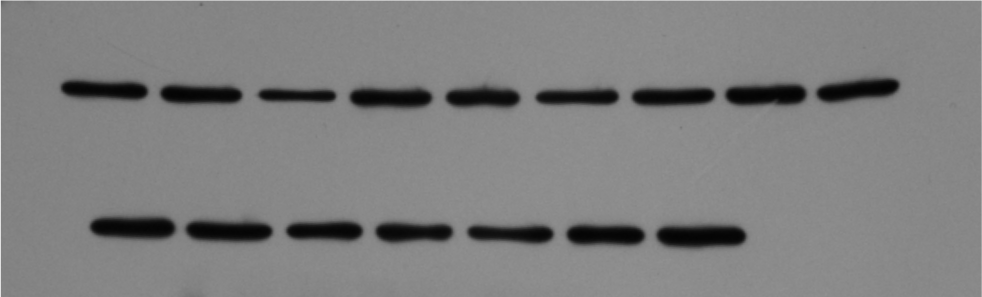

Supplement: Supplementary file 4 [file Image_3.TIF]

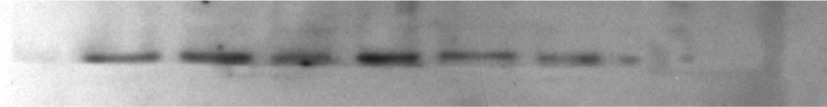

Supplement: Supplementary file 5 [file Image_4.TIF]

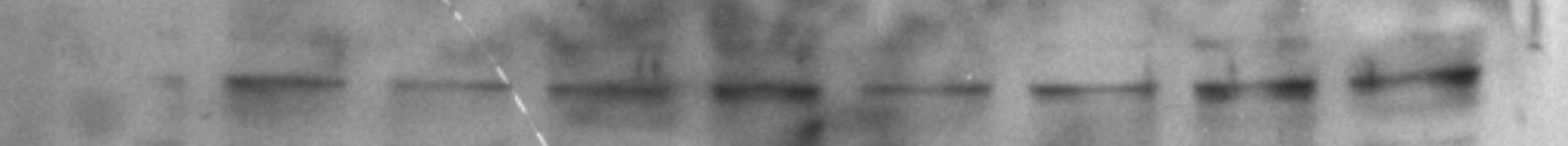

Supplement: Supplementary file 6 [file Image_5.TIF]
